# Colonization of Three Maple Species by Asian Longhorned Beetle, *Anoplophora glabripennis*, in Two Mixed-Hardwood Forest Stands

**DOI:** 10.3390/insects5010105

**Published:** 2013-12-31

**Authors:** Kevin J. Dodds, Helen M. Hull-Sanders, Nathan W. Siegert, Michael J. Bohne

**Affiliations:** 1 Northeastern Area State and Private Forestry, U.S. Department of Agriculture Forest Service, 271 Mast Rd., Durham, NH 03824, USA; E-Mails: nwsiegert@fs.fed.us (N.W.S.); mbohne@fs.fed.us (M.J.B.); 2 Otis Laboratory, Center for Plant Health Science and Technology, U.S. Department of Agriculture, 1398 W. Truck Rd., Buzzards Bay, MA 02452, USA; E-Mail: Helen.M.Hull-Sanders@aphis.usda.gov

**Keywords:** host suitability, Cerambycidae, *Acer*, host selection, invasive species

## Abstract

Asian longhorned beetle (ALB), *Anoplophora glabripennis* (Motschulsky), is an invasive insect that has successfully established multiple times in North America. To investigate host colonization and reproductive success (exit holes/eggs), two ALB infested forest stands were sampled in central Massachusetts, USA. Infested *Acer platanoides* L., *Acer rubrum* L., and *Acer saccharum* Marsh. were felled, bucked into 1 m sections and dissected to determine indications of ALB infestations, such as presence of life stages or signs of damage on trees. ALB damage was also aged on a subset of trees to determine the earliest attacks on the three *Acer* species. In one stand, ALB oviposition was significantly higher on the native *A. rubrum* and *A. saccharum* than the exotic *A. platanoides*. In the second stand, ALB oviposition was significantly higher and cumulative reproductive success was higher on *A. rubrum* than *A. platanoides* or *A. saccharum*. An *A. saccharum* had the earliest signs of attack that occurred in 2006. *Acer rubrum* (2007) and *A. platanoides* (2010) were colonized shortly thereafter. Overall, ALB was more successful in *A. rubrum*, where adults emerged from 53% and 64% of trees in each stand, compared to *A. platanoides* (11% and 18%) or *A. saccharum* (14% and 9%).

## 1. Introduction

The Asian longhorned beetle (ALB), *Anoplophora glabripennis* (Motschulsky) (Coleoptera: Cerambycidae), has successfully invaded urban forests in North America and Europe [1]. In North America, ALB has become established in several cities and townships, including New York, New York; Chicago, Illinois; Jersey City and Carteret, New Jersey; Toronto, Ontario; Worcester and Boston, Massachusetts and Bethel, Ohio [2,3]. The infestation in Worcester, Massachusetts is the largest detected in North America thus far, and as of December 2013 has resulted in a 285 km^2^ quarantine zone and over 33,000 trees removed during the ongoing eradication effort. Most recently, an ALB infestation was detected in Tate Township, Ohio, a rural area approximately 48 km east of Cincinnati. The Ohio and Massachusetts infestations are both adjacent to large areas of forests with abundant ALB host tree species, making these established populations particularly worrisome as they could provide a source for dispersal into forests of the region if not contained. ALB populations in Illinois and New Jersey have been successfully eradicated, while efforts to eliminate the beetle from other areas are ongoing.

ALB is native to China and the Korean Peninsula where it was generally considered to be innocuous until large plantations of exotic *Populus* matured and provided extensive areas of suitable habitat for the insect to exploit [1]. While ALB most often damaged *Populus* in China, other hardwood trees were also colonized [1]. However, even with the economic damage caused in parts of its native range, little is known about ALB behavior in natural forests. Unlike China, epidemic ALB populations have not been recorded in South Korea. ALB populations in South Korea also exhibit behavioral differences compared to populations in China, such as a preference to colonize *Acer* spp. [4]. Williams *et al*. [4] speculated that ALB is an edge specialist in riparian habitats and dispersal into interior forests is limited in Korea. However, relatively large dispersal distances (>1 km) have been recorded for male and female ALB in China during field-based mark-recapture experiments [5].

Forests of eastern North America are tree species rich and provide plentiful hosts, such as *Acer* spp., *Aesculus* spp., *Betula* spp., *Ulmus* spp., and *Salix* spp. for a polyphagous feeder like ALB. Through work in urban forest infestations in North America and common garden studies in Asia, a list of common tree genera and their suitability for ALB exists and has been used in management activities [6]. In an urban-based infestation, a greater proportion of *Acer* and *Ulmus* were infested compared to other host trees [7]. Laboratory studies have provided further insight into the suitability of North American trees for ALB development and have suggested that *Acer* is a preferred genus. Logs of *Acer platanoides* L. had significantly more oviposition than *Acer rubrum* L. or *Salix nigra* Marsh. in a no-choice study [8]. In another study, ALB larval weight gain was compared among commonly encountered urban trees with results suggesting *Ulmus* and *Acer* provided the best habitat for growth from those tested [9]. Higher numbers of oviposition pits and larvae were found on *Acer saccharum* Marsh. compared to *A. rubrum*, *Fraxinus pennsylvanica* Marsh., or *Quercus rubra* L. in a greenhouse study [10]. While previous studies and observations during survey suggest ALB may either oviposit more often on, or perform better on *Acer*, there are currently no published studies comparing ALB survivorship and reproductive success in naturally colonized *Acer* growing in forested environments.

Prior to the infestation in Worcester, Massachusetts, ALB infestations in North America had been confined to urban areas. ALB has now been found in several forest stands in and around Worcester, providing opportunities to collect information on behavior in forested areas before eradication efforts commence. Sampling from infested forest stands in Worcester documented that *Acer* spp. were the only trees attacked by ALB during early stages of forest invasion even though other host genera were present [11]. In terms of specific *Acer* species, *A. rubrum* was attacked more frequently than *A. platanoides* or *A. saccharum* but it is unknown if *A. rubrum* was also more intensively colonized by ALB compared to the other *Acer* spp. present. The objective of this study was to investigate ALB attack densities, densities of different life stages, and cumulative reproductive success among three forest grown *Acer* spp. in Massachusetts hardwood forests. Knowledge of ALB success in forest trees is critical to understanding potential impacts, enhancing survey protocols in forested settings, and developing future management techniques.

## 2. Materials and Methods

**Figure 1:**
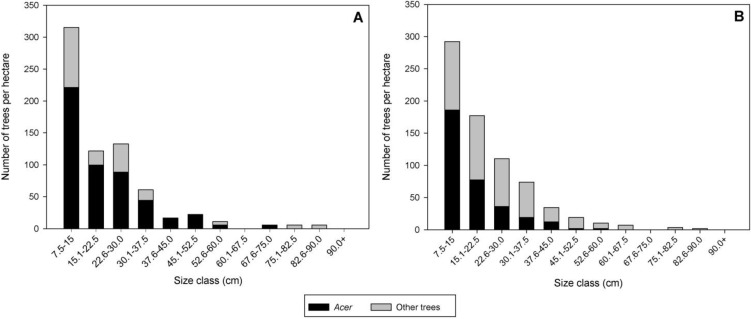
Distribution of *Acer* spp. and other hardwood trees with diameter at breast height (DBH) ≥ 7.5 cm among size classes of DBH in Delaval (**A**) and Boylston (**B**).

*Site descriptions*: We sampled two stands infested with ALB from the Worcester Massachusetts quarantine area. The Delaval stand (N 42.3120°, W −71.8089°) was identified as infested in fall 2009 and sampled from December 2009 to January 2010. Delaval was an approximately 5 ha *A. rubrum*-dominated stand that also contained *A. saccharum* and *A. platanoides* in the overstory. *Acer rubrum* accounted for 52% of the relative basal area, while *A. platanoides* (7%) and *A. saccharum* (4%) contributed less. *Acer* spp. were spread throughout the stand, with some pockets of *A. platanoides* concentrated along the eastern edge of the stand. Approximately 63% of *Acer* trees were infested by ALB in Delaval [11]. The stand was uneven-aged, with *Acer* present in most size classes (Figure 1A). Overstory trees present in the stand included *Q. rubra*, *Quercus velutina* Lam., *Fraxinus americana* L., *Betula lenta* L., and several other species. A complete stand description of Delaval was previously published [11]. All host species were removed from this stand in winter 2010.

In the second stand, Boylston (N 42.3276°, W −71.7507°), ALB were detected in the summer 2010 and trees were sampled in November of the same year. The portion of the stand in Boylston where trees were selected for sampling was approximately 10 ha and had been surveyed by ALB program tree climbers. No prior vegetation survey had been conducted in Boylston, so 13 fixed radius plots (452 m^2^) were established throughout this 10 ha area to describe the forest stand. Protocols followed Dodds and Orwig [11], with tree species, diameter at breast height (DBH), crown class, living-dead, and ALB presence/absence recorded for every tree ≥7.5 cm DBH. All host species were removed from this stand shortly after sampling.

**Figure 2:**
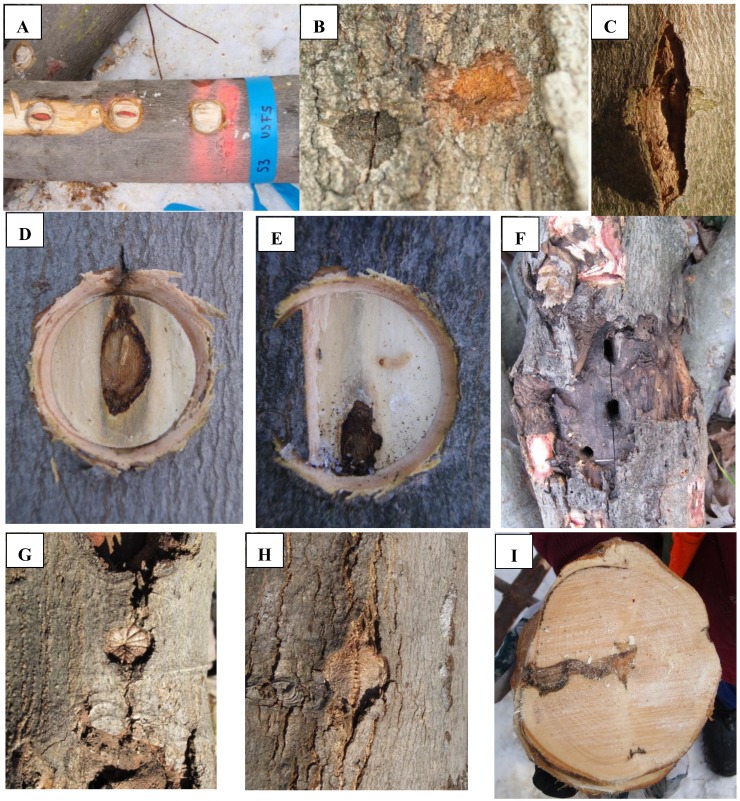
Log section with bark removed from oviposition pits (**A**). Older (left) and recent (right) Asian longhorned beetle (ALB) oviposition pits on infested maple (**B**). Calloused ALB oviposition pit (**C**). Dissection of oviposition pit showing egg (**D**). Dissected oviposition pit with ALB larva and indications of larval feeding (**E**). ALB late larvae sapwood entrance holes (oval holes, center) and an exit hole (bottom, left) (**F**). Calloused ALB exit hole (**G**,**H**). Infested tree cross section following ALB development through to an exit hole (**I**).

*Tree selection—life stages*: ALB host trees in both stands were surveyed by ALB program tree climbers. Climbers searched boles and crowns on trees and marked trees as ALB positive if an oviposition site, bark eruption, or exit hole was located (Figure 2). Trees with no indications of ALB attack or colonization were marked as uninfested. Stands were carefully examined for signs of tree mortality that could be attributed to ALB. From the available ALB-infested trees, a subset of the three *Acer* species present was selected for use in intensive sampling efforts. Random selection of infested *Acer* occurred in Boylston where there were more infested trees present than our goal of ten replicates for each *Acer* species. Due to the relatively low numbers of *A. saccharum* and *A. platanoides* present in the Delaval stand, the selection of these two species was not random and every available infested tree was sampled. Because higher numbers of infested *A. rubrum* were present in Delaval, we were able to randomly select replicates of this tree species. The majority (58%) of trees sampled were from the codominant crown class, with fewer trees sampled in intermediate or overtopped classes.

Thirty-three trees were selected for intensive sampling from each stand. Seventeen *A. rubrum*, seven *A. saccharum*, and nine *A. platanoides* were sampled from Delaval. Eleven each of *A. rubrum*, *A. saccharum*, and *A. platanoides* were sampled from Boylston. Selected trees were felled and two height measurements recorded: (1) total tree length, the distance from the base of tree to the tip of the live crown; and (2) bole length, the distance from the base of tree to the base of live crown. Length of live crown was then calculated by subtracting bole length from total tree length. The bole was cut into 1 m long log sections. Diameter at midpoint of each 1 m section was measured using calipers. The live crown was often cut into smaller sections to facilitate sampling, but data were pooled for analysis. Tree characteristics are detailed in Table 1.

*Damage age on infested trees*: In addition to the 33 trees sampled from Boylston, seven *A. rubrum*, nine *A. platanoides*, and 13 *A. saccharum* were felled and examined for “old damage”. Old damage was defined as ALB oviposition pits or exit holes where some level of callous tissue had formed or another sign or symptom indicated relatively older damage. Sections of tree boles with the oldest apparent damage were removed from trees for further dissection under laboratory conditions. Samples were taken to USDA APHIS PPQ Center for Plant Health Science and Technology, Otis Laboratory, Buzzards Bay, Massachusetts and dried in an ESPEC Platinous Sterling temperature and humidity chamber at 80 °C overnight. Log sections were removed from the chamber and allowed to rest for ≥24 h. Calloused ALB exit holes or oviposition pits were identified, removed by band saw from the larger log section, sanded using a belt sander and examined under the microscope to determine the number of tree growth rings since initial damage had occurred. This provided an accurate estimate of when the tree was infested or when a cohort emerged.

**Table 1:** Mean (±SE) diameter, total height, bole length, and crown length of trees sampled for Asian longhorned beetle in Delaval (2009) and Boylston (2010).

Species	N	Mean Diameter (cm)	Mean total height (m)	Mean bole length (m)	Mean crown length (m)
**Delaval**					
*Acer rubrum*	17	17.0 ± 1.2	15.5 ± 0.9	7.3 ± 0.5	8.2 ± 0.7
*Acer saccharum*	7	18.6 ± 1.9	13.5 ± 1.5	6.7 ± 0.8	6.8 ± 1.1
*Acer platanoides*	9	21.9 ± 1.7	15.1 ± 1.3	7.8 ± 0.7	7.3 ± 1.0
**Boylston**					
*Acer rubrum*	11	19.2 ± 2.3	17.4 ± 1.2	8.2 ± 0.9	9.2 ± 0.8
*Acer saccharum*	11	20.5 ± 2.3	20.3 ± 1.2	10.7 ± 0.9	9.5 ± 0.8
*Acer platanoides*	11	20.1 ± 2.3	18.9 ± 1.2	10.5 ± 0.9	8.3 ± 0.8

Note: No significant differences (*p* > 0.05) among the three *Acer* species were found in Delaval and Boylston.

*Life stage sampling*: Sampling methods varied slightly between the two ALB infested stands. In Delaval, four indicators of ALB presence were defined (oviposition pit, early larva, late larva, and exit hole), while five were defined in Boylston (oviposition pit, egg, early larva, late larva, and exit hole). Severe winter temperatures made sampling eggs difficult and inconsistent in Delaval. Thus, egg estimates were not included in any analyses from this stand. All 1 m log sections and each tree crown were searched for indicators of ALB presence (Figure 2A). ALB re-attacks brood trees over multiple years [12,13], therefore, sample trees generally contained evidence from multiple cohorts. Consequently, a combination of ALB life stages (*i.e.*, egg, early larvae, and late larvae) and historical evidence (*i.e.*, oviposition pits, phloem/sapwood staining, sapwood entrance holes, and exit holes) were used to estimate life stage densities over the entire period trees were attacked. The cumulative number of each ALB sign or life stage was recorded for each log section.

ALB oviposition pits (Figure 2B) are generally distinct oval pits chewed through the bark into the phloem, but can also be much smaller vertical slits through thinner bark areas of trees [12]. Older oviposition pits are often swollen or erupted areas on the bark caused by the wound response and lack the characteristic shape often used for ALB diagnosis (Figure 2C). However, mandible marks made during pit excavation were still present and usually discernible. In this study, bark was carefully examined for oviposition pits and when encountered each one was tallied and then removed with a chisel or hatchet to expose the phloem. Pits were then examined for the presence of an egg, a distinct stain associated with an egg in older pits [8] living or dead early larvae, or evidence of larval feeding activity in the oviposition pit area (Figure 2D,E).

Early larvae were defined behaviorally as larvae that had only fed in the phloem/sapwood interface and had not horizontally entered the sapwood. If frass was present directly under the oviposition pit or, in older pits, a larger area of staining and/or gallery occurred, this indicated the presence of an early larva (Figure 2E). Often older oviposition pits (3 + years) did not contain frass, but staining in the shape of larval-feeding galleries was present indicating an early larva had been present under the oviposition pit.

Late larvae were differentiated from early larvae by the presence of an oval sapwood entrance hole in the phloem/sapwood feeding gallery (Figure 2F). ALB larvae pass through several instars in the phloem before entering the sapwood where they complete development [12]. These sapwood entrance holes are distinct and easily recognizable based on their shape and location within a feeding gallery.

The presence of exit holes indicated complete development and emergence of ALB in a section of tree. Exit holes varied considerably from obvious round holes exiting the sapwood to older calloused wounds (Figure 2G,H). For any suspect exit hole that was calloused, log sections were further sectioned into small “cookies” to follow the wound (Figure 2I). Calloused round holes that connected to or aligned with sapwood galleries were then tallied as exit holes for the log section.

When feeding activity obscured evidence of specific life stages, counts were estimated based on a combination of life stage presence and expert opinion. No attempt was made to differentiate the age of ALB evidence by year and all counts were combined into one estimate of cumulative life stage densities for the signs and life stages. Traditional life table analysis was not appropriate due to the inability to differentiate among cohorts within a tree. For trees in the Boylston stand, an estimate of cumulative reproductive success for each tree was determined by dividing number of exit holes by number of eggs in each tree. Cumulative reproductive success could not be estimated because eggs were not counted from all trees in Delaval.

*Statistical analyses*: The two stands were analyzed separately given the unique conditions present in each forest. Tree variables (DBH, total tree height, bole length, and crown length) were compared among species with a generalized linear mixed model (PROC GLIMMIX) via maximum likelihood estimation technique (SAS Institute Inc., v. 9.3, 2011, Cary, NC, USA). Because the dependent data were continuous and normally distributed, the normal distribution and identity link were used for analysis. Estimates of life stage densities were summed by tree for inter-tree comparisons. The mean number of oviposition pits, eggs, early larvae, late larvae, and exit holes per tree were also analyzed using a generalized linear mixed model (PROC GLIMMIX) via maximum likelihood estimation technique. Tree species was a fixed effect, and because data were non-normal, the negative binomial distribution with log link was used for analysis. Mean differences for all tests were separated using Tukey’s HSD. Cumulative reproductive success was not statistically analyzed due to the large number of zero counts in the dataset. Mean and standard errors (SE) are reported for cumulative reproductive success from the Boylston stand.

## 3. Results

*Delaval—2009*: Mean diameter (*F* = 2.8, d.f. = 2, 30; *p* = 0.07), total tree height (*F* = 0.7, d.f. = 2, 30; *p* = 0.5), bole length (*F* = 0.6, d.f. = 2, 30; *p* = 0.6), and crown length (*F* = 0.7, d.f. = 2, 30; *p* = 0.5) were not statistically different among the three *Acer* species (Table 1), allowing for direct comparisons of life stage estimates among the tree species. Of the 33 trees categorized as positive for ALB by tree climbers, evidence of an ALB life stage or damage was located on every tree during dissection. No tree mortality was observed.

**Figure 3:**
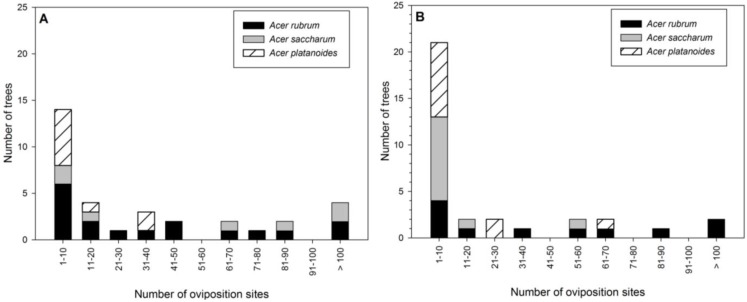
Number of Asian longhorned beetle oviposition pits per tree by *Acer* species in Delaval (**A**) and Boylston (**B**).

**Figure 4:**
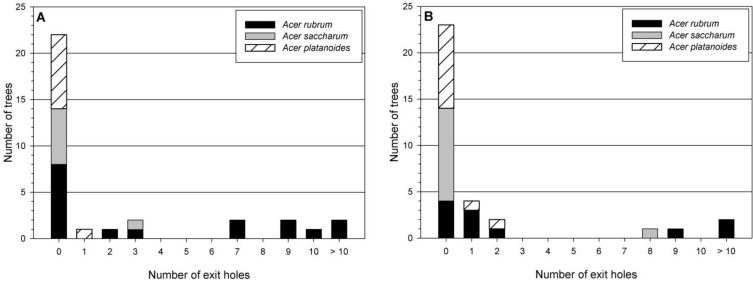
Number of Asian longhorned beetle exit holes per tree by *Acer* species for sampled trees in Delaval (**A**) and Boylston (**B**).

The number of oviposition pits on trees ranged from 1 to 208, but the majority of trees had few attacks (Figure 3A). While none of the *A. platanoides* sampled had >40 oviposition pits, seven of the 17 *A. rubrum* and four of the seven *A. saccharum* sampled had >40 oviposition pits (Figure 3A). *Acer rubrum* had 73% and *A. saccharum* had 83% more oviposition pits compared to *A. platanoides* (*F* = 4.4, d.f. = 2, 30; *p* = 0.02; Table 2). The number of oviposition pits on *A. rubrum* and *A. saccharum* were not statistically different. Early larvae (*F* = 4.2, d.f. = 2, 30; *p* = 0.03) followed a similar pattern to oviposition pits, with significantly more found on *A. rubrum* and *A. saccharum* compared with *A. platanoides* (Table 2). There were significantly more late larvae (*F* = 9.3, d.f. = 2, 30; *p* = 0.0007) and exit holes (*F* = 6.0, d.f. = 2, 30; *p* = 0.006) per tree in *A. rubrum* than *A. saccharum* and *A. platanoides* (Table 2). Over 85% of individual *A. saccharum* and *A. platanoides* trees produced no adults, while 47% of *A. rubrum* did not (Figure 4A).

**Table 2:** Mean (±SE) estimates of life stages and signs of Asian longhorned beetle per tree in three *Acer* species from the Delaval stand. Means followed by the same superscript letter within a row are not significantly different (Tukeys HSD, *p* > 0.05).

Variable	*Acer rubrum*	*Acer saccharum*	*Acer platanoides*	*p*-value
Oviposition pits	44.5 ± 13.9 ^a^	70.9 ± 34.5 ^a^	11.9 ± 5.2 ^b^	0.02
Early larvae	21.8 ± 7.3 ^a^	20.3 ± 10.7 ^a^	4.2 ± 2.1 ^b^	0.03
Late larvae	6.3 ± 2.0 ^a^	1.0 ± 0.6 ^b^	0.3 ± 0.2 ^b^	0.0007
Exit holes	4.1 ± 1.8 ^a^	0.4 ± 0.4 ^b^	0.1 ± 0.1 ^b^	0.006

*Boylston—2010*: There were 723 stems/ha and 35.3 m^2^/ha of basal area distributed among 14 tree species in the Boylston stand (Table 3). *Acer saccharum* represented 24% of the stand basal area and had the highest relative importance value (30.8) followed by *F. americana* (19.9), *Q. velutina* (13.5), and *Q. rubra* (12.2). This stand was uneven-aged and contained host trees in multiple size classes (Figure 1B). The ALB infestation was not as extensive in Boylston as in Delaval with approximately 14% of *Acer* host trees infested.

Average diameter (*F* = 0.09, d.f. = 2, 30; *p* = 0.9), total tree height (*F* = 1.32, d.f. = 2, 30; *p* = 0.3), bole length (*F* = 2.3, d.f. = 2, 30; *p* = 0.1), and crown length (*F* = 0.6, d.f. = 2, 30; *p* = 0.5) were not statistically different among the three *Acer* species (Table 1). Of the 33 trees categorized as positive for ALB, all had evidence of an ALB life stage or sign present. No tree mortality was observed.

**Table 3:** Mean diameter, basal area, density, and importance values for trees sampled in Boylston.

Tree Species	Mean ± SE DBH (cm)	Basal area (m^2^/ha)	Stems /ha	Relative basal area (%)	Relative density (%)	Importance value **
*Acer saccharum* *	17.8 ± 0.7	5.0	159	24. 3	37.4	30.8
*Fraxinus americana*	21.2 ± 0.8	3.7	93	17.9	21.9	19.9
*Quercus velutina*	31.8 ± 1.6	3.6	41	17.3	9.6	13. 5
*Quercus rubra*	37.3 ± 3.3	3.7	28	17.9	6.6	12.2
*Pinus strobus*	36.1 ± 5.2	2.3	17	11.1	4.0	7.6
*Acer rubrum* *	13.8 ± 1.4	0.5	28	2.6	6.6	4.6
*Ulmus* sp. *	11.3 ± 0.7	0.3	30	1.6	7.1	4.3
*Quercus alba*	31.7 ± 5.4	1.1	11	5.4	2.6	4.0
*Fagus grandifolia*	25.4 ± 5.4	0.2	4	1.1	0.9	1.0
*Acer platanoides* *	11.0 ± 1.3	0.07	7	0.3	1.6	1.0
*Juglans nigra*	15.1 ± 0.1	0.04	2	0.2	0.5	0.3
*Malus* sp.	12.0 ± 0.4	0.02	2	0.1	0.5	0.3
*Ostrya virginiana*	9.6 ± 1.0	0.01	2	0.07	0.6	0.3
*Betula populifolia* *	8.6 ± 0.0	0.005	1	0.03	0.2	0.1
Total		20.5	425			
Total/ha		35.3	723			

* Tree genus listed as preferred host in the USA [6]; ** Importance Value = (relative basal area + relative density)/2.

**Figure 5:**
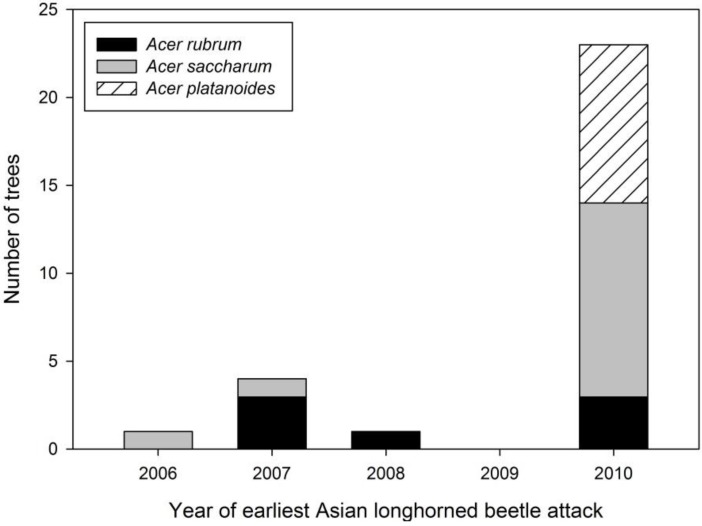
Distribution of age of earliest Asian longhorned beetle attack by *Acer* species in Boylston.

The earliest evidence of ALB colonization was found on the two native *Acer* species (Figure 5). An exit hole on an *A. saccharum* was aged to 2007, suggesting it was first successfully colonized in 2005 or 2006 depending on generation time. Infestation dates ranged from 2007–2010 for *A. rubrum* with evidence of both exit holes (2008) and oviposition pits. All sampled *A. platanoides* were found to have only oviposition pits dated to 2010.

**Figure 6:**
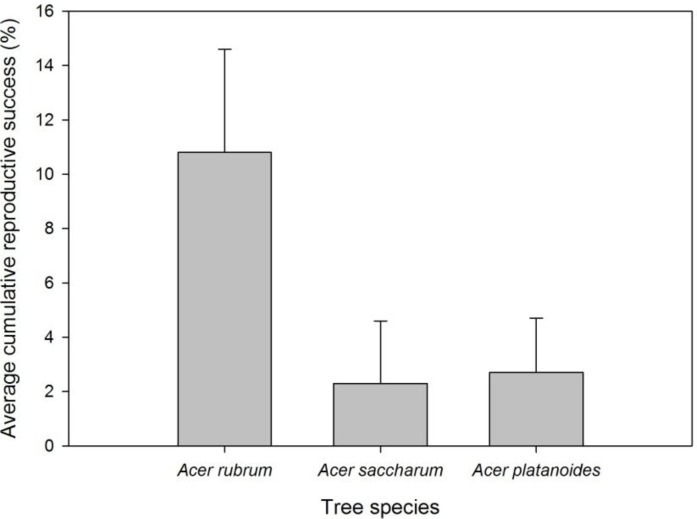
Mean cumulative reproductive success of Asian longhorned beetle by tree species in Boylston. Reproductive success was determined by dividing the number of eggs by exit holes.

The number of oviposition pits ranged from 1 to 340, with the majority of trees having between 1 and 10 (Figure 3B). Only one each of *A. platanoides* and *A. saccharum* had >50 oviposition pits (Figure 3B). Significantly more oviposition pits (*F* = 9.4, d.f. = 2, 30; *p* = 0.0007), eggs (*F* = 10.5, d.f. = 2, 30; *p* = 0.0003), and early larvae (*F* = 8.8, d.f. = 2, 30; *p* = 0.001) per tree were found on *A. rubrum* compared to *A. saccharum* or *A. platanoides* (Table 4). Percentage of oviposition pits with eggs varied in Boylston, with *A. rubrum* (89%) and *A. platanoides* (75%) much higher than *A. saccharum* (24%). *Acer saccharum* had a similar number of sapwood entrance holes as *A. rubrum* and *A. platanoides*, while *A. rubrum* had higher numbers than *A. platanoides* (*F* = 4.4, d.f. = 2, 30; *p* = 0.02; Table 4). More exit holes were found in *A. rubrum* per tree compared to *A. saccharum* and *A. platanoides* (*F* = 4.5, d.f. = 2, 30; *p* = 0.02; Table 4). Most *A. saccharum* (91%) and *A. platanoides* (82%) produced no adults, while only 36% of *A. rubrum* did not (Figure 4B). On average, almost four times the number of adults were produced from *A. rubrum* than *A. saccharum* or *A. platanoides* (Figure 6).

**Table 4:** Mean (±SE) estimates of life stages and signs of Asian longhorned beetle in three *Acer* species from the Boylston stand. Means followed by the same superscript letter within a row are not significantly different (Tukeys HSD, *p* > 0.05).

Variable	*Acer rubrum*	*Acer saccharum*	*Acer platanoides*	*p*-value
Oviposition pits	65.3 ± 21.5 ^a^	9.3 ± 3.2 ^b^	14.4 ± 4.8 ^b^	0.0007
Eggs	55.0 ± 21.9 ^a^	3.9 ± 1.7 ^b^	11.7 ± 4.8 ^b^	0.0003
Early larvae	48.6 ± 21.9 ^a^	3.2 ± 1.5 ^b^	9.6 ± 4.4 ^b^	0.001
Late larvae	14.7 ± 8.2 ^a^	3.0 ± 1.7 ^a,b^	1.4 ± 0.8 ^b^	0.02
Exit holes	6.7 ± 4.7 ^a^	0.7 ± 0.6 ^b^	0.3 ± 0.2 ^b^	0.02

## 4. Discussion

ALB is an effective invader that has established populations outside of its native range in North America and Europe on various hardwood tree species [2]. Repeated detections of ALB in urban settings of North America have raised concerns for this species becoming permanently established in hardwood forests. Estimated costs for ALB impacts in the U.S. have been as high as $669 billion [14]. While eradication efforts attempt to eliminate current populations in Massachusetts and elsewhere, it is important to gather information on ALB behavior in North American forests when opportunities arise. To date, these opportunities have been limited in forested settings. The two stands sampled in this study were dominated by different *Acer* species and represented important forest types in the region. While sampling in these two stands provided important information, care must be taken in drawing broader conclusions on ALB behavior and possible impacts based solely off of results from a limited sample.

*Stand structure*: The two stands sampled for this study were mixed hardwood stands with *Acer* and other tree species present in the overstory. Both stands contained *A. platanoides*, *A. rubrum*, and *A. saccharum* and these trees were the only species attacked by ALB at the time of discovery. Stand structure in Boylston varied slightly from those previously recorded for Delaval [11]. Boylston was dominated by *A. saccharum*, with *F. americana* and *Quercus* spp. as important overstory species. *Acer platanoides* and *A. rubrum* had relatively low importance values in Boylston. In comparison, Delaval was dominated by *A. rubrum*, with *Quercus* spp. as important overstory components. *Acer platanoides* was a relatively important tree species in Delaval. Both stands contained non-*Acer* ALB hosts, including *Betula* spp. and *Ulmus* sp.

*Age of attacks*: Timing of attack may confound comparisons of ALB life stages on the three *Acer* species. Age-related damage estimates were only collected in Boylston. These data provide some insight into ALB colonization of the three hosts. The earliest indication of ALB presence in Boylston was found on an *A. saccharum* with an exit hole that occurred in 2007 suggesting the tree was first colonized in 2005 or 2006 depending on generation time in Massachusetts. Anecdotal evidence observed during tree dissections suggested a two-year life cycle may be occurring in Massachusetts (K.J.D., personal observation), but it is unknown how common this is. While data suggest that *A. saccharum* was the first tree colonized in the stand, the majority of this tree species were first colonized in 2010. Conversely, the majority (57%) of *A. rubrum* were attacked in 2007–2008, with remaining trees first attacked in 2010. *Acer platanoides* that were aged were not colonized until 2010. However, two trees dissected for life stages contained recent exit holes, suggesting these trees were attacked in 2008 or 2009.

Unfortunately, due to destructive sampling for the age determination and life stage sampling and working under the constraints of an impending eradication cut, very few trees were used for both data sets. Consequently, some age related data may have been lost during tree dissections and *vice versa*. While the data that were generated for the aging provides some information on ALB colonization patterns in Boylston, it does not provide a complete picture of the infestation pattern. The sequence of attacks on *Acer* species is an important issue when considering ALB reproductive estimates among host trees as it appears that asynchronous colonization of the three tree species occurred. More *A. rubrum* were colonized for a longer period of time than *A. saccharum* or *A. platanoides* even though *A. saccharum* was the first tree colonized in the stand. Detailed examination of ALB host selection and spatial attack pattern in forested environments is needed.

*Life stage comparisons*: For the experimental trees, diameter, total height, bole height, and crown length of the three *Acer* spp. at both sites were similar suggesting that available resources for ALB were equal among the tree species. However, there was variation in the average number of oviposition pits among the three *Acer* spp. examined in our study. *Acer rubrum* was consistently one of the tree species with the most oviposition pits in both stands. This is counter to previous studies that found ALB oviposition was greater on *A. platanoides* [8] and *A. saccharum* [10] compared to other hosts, including *A. rubrum* in laboratory studies.

Asynchronous host colonization could partially explain the higher abundance of oviposition pits on *A. rubrum*. However, *A. saccharum* was at least initially attacked by ALB before the other host species. In the two years following the attack on *A. saccharum*, ALB colonization apparently shifted to *A. rubrum*. *Acer platanoides* was colonized later than the two native species, but had statistically the same number of pits as *A. saccharum*, a species with a longer attack history. It is unknown why the two native *Acer*, and especially *A. rubrum*, frequently had higher numbers of ovipositon pits, but differences in host defenses [15] or volatile profiles [16] among the trees may be factors.

In terms of life stages sampled, *A. rubrum* generally had greater evidence of beetle presence than *A. saccharum* or *A. platanoides*. While *A. rubrum* was always statistically separated from *A. platanoides*, *Acer saccharum* often fell somewhere in between life stage estimates of the two other *Acer* species. Equal numbers of early larvae were found on *A. saccharum* and *A. rubrum* in Delaval, but only similar numbers of late larvae in Boylston. All other life stage estimates were lower in *A. saccharum* than *A. rubrum*. In addition, the percentage of oviposition pits with eggs was lowest on *A. saccharum*, suggesting that this species might be tested by ALB, but not chosen as a reproductive resource as often as *A. rubrum*.

Evidence of multiple generations (e.g., new and old oviposition pits, calloused exit holes, and larval galleries in various conditions) was present in sampled trees and was not temporally differentiated. Consequently, age-specific survivorship could not be determined from this data set and care must be taken in drawing conclusions on stage-specific survivorship or mortality factors. High stage-specific mortality has been noted in ALB larvae developing in *Populus* from Chinese infestations [17]. A similar pattern of high larval mortality was observed in this study. In both stands, it appears that significant mortality occured between the early to late larvae development period. Further study into possible causal agents of this mortality could be beneficial to gaining insights into ALB population regulation mechanisms. The high levels of larval mortality may explain the lack of host tree mortality noted in both stands.

While it was not possible for cumulative reproductive success estimates to be statistically compared because of the large numbers of zeros in the data set, some insights into ALB success in the three *Acer* spp. were apparent. First, reproductive success was low in *A. platanoides*, suggesting this species is a poor host for ALB in forests or a suboptimal host early in the invasion process. This finding differs from previous work in which *A. platanoides* was considered an intermediate host species in an urban infestation [7]. In laboratory studies, Smith *et al*. [8] evaluated oviposition on cut bolts under a no-choice experimental design and reported higher daily and lifetime oviposition by ALB on *A. platanoides* compared to *A. rubrum*. Studies conducted in a forested environment, as reported herein, indicate that *A. platanoides* is less suitable for completion of development from egg to adult than *A. rubrum*, at least at the beginning of colonization. Second, reproductive success was high in *A. rubrum* in Boylston, providing evidence that this species is a suitable host as well as producing relatively greater numbers of adults. Reproductive success in *A. saccharum* was much lower than *A. rubrum* in Boylston, but in one tree the number of adult exit holes was relatively high, indicating this tree species is good habitat for ALB.

## 5. Conclusions

Information from both stands implied that the geographically and ecologically widespread *A. rubrum* is a good host tree for ALB. Not only did higher numbers of adults emerge from *A. rubrum* compared to other *Acer* spp. present in the stand, but also successful reproduction from egg to adult happened more often on this tree. Fifty-three percent and 64% of *A. rubrum* in Delaval and Boylston, respectively, produced at least one adult per tree. In comparison, only 14% and 9% of the *A. saccharum* produced adults in Delaval and Boylston, respectively. A similar pattern was found for *A. platanoides*, where only 11% and 18% of the sampled trees produced adults in Delaval and Boylston, respectively. *Acer rubrum* was attacked more frequently than other *Acer* spp. [11] and results from this study suggest that it is often attacked more intensively as well, even when it is not a dominant tree in a stand.

Given that large areas of eastern North America are at risk to ALB invasion [18], various state and federal agencies expend considerable resources surveying high risk environments for this insect. Gathering as much data as possible from infested stands is important for providing information that may be helpful to the eradication program or detection surveys outside of regulated areas. Working within the confines of a large eradication program is often difficult, but data collected from these opportunities is critical for gaining a better understanding of invasive species behavior in native forests. ALB detection surveys generally consist of ground-based efforts often with binoculars, bucket trucks to access tree crowns, and/or professional tree climbers [12]. Outside of quarantine areas where all known host trees are surveyed, high risk sites such as high visitation campgrounds where recreationists may have moved firewood, areas adjacent to businesses importing large quantities of material, or port areas where introductions could occur are often prioritized for survey. However, within these high risk sites information on how to prioritize survey is generally lacking with the exception of focusing on known host species. It is unclear from this work if *A. rubrum* is a more suitable host than other *Acer* species or if it is generally colonized before other host species. While other *Acer* spp. should not be ignored during survey efforts, extra attention to *A. rubrum* may be beneficial for determining ALB presence in an area.
